# Aerobic anti‐gravity exercise in patients with Charcot–Marie–Tooth disease types 1A and X: A pilot study

**DOI:** 10.1002/brb3.794

**Published:** 2017-11-02

**Authors:** Kirsten L. Knak, Linda K. Andersen, John Vissing

**Affiliations:** ^1^ Copenhagen Neuromuscular Center Department of Neurology Rigshospitalet University of Copenhagen Copenhagen Denmark

**Keywords:** anti‐gravity, Charcot–Marie–Tooth disease, exercise

## Abstract

**Background:**

Charcot–Marie–Tooth (CMT) disease is a hereditary neuropathy associated with impaired walking capacity. Some patients are too weak in the lower extremity muscles to walk at gravity with sufficient intensity or duration to gain benefit.

**Aim:**

The aim was to investigate the effect of aerobic anti‐gravity exercise in weak patients with CMT 1A and X.

**Methods:**

Five adult patients performed moderate‐intensity aerobic anti‐gravity exercise 3/week for 10 weeks.

**Results:**

There was a significant positive difference in Berg balance scale and postural stability test between test occasions, and walking distance in the 6‐min walk test trended to increase.

**Conclusions:**

The study indicates that the anti‐gravity treadmill training of patients with CMT should be pursued in larger CMT cohorts.

## 1 INTRODUCTION

Charcot–Marie–Tooth (CMT) disease is a hereditary neuropathy affecting motor and sensory nerves. CMT type 1A and X present similar phenotypes and are slowly progressive, demyelinating diseases characterized by distal muscle weakness, sensory loss, and joint deformities. There is no effective treatment for CMT (Jani‐Acsadi, Krajewski, & Shy, 2008), but exercise is promising (Sman et al., 2015). Effect of walk training has been investigated in CMT (Maggi et al., 2011; Wright et al., 1996), but some patients are too severely affected to walk with gravity at sufficient intensity or duration to obtain benefit. This is possible on an anti‐gravity treadmill, which off lifts load on the lower extremities.

The objective was to investigate the effect of aerobic anti‐gravity exercise in moderately to severely walking disabled patients with CMT 1A and X.

## 2 METHODS

### 2.1 Patients

Patients were recruited from our neuromuscular center between 2015 and 2016. Inclusion criteria were as follows: (1) genetically verified CMT type 1A or X, (2) ≥18 years, and (3) walking ability ≥10 m. Exclusion criteria were as follows: (1) compliance issues, (2) medical conditions that could contraindicate physical exercise, (3) walking ability >450 m in a 6‐min walk test (6MWT), (4) pregnancy, (5) pacemaker, (6) aerobic exercise >1 hr/week, and (7) long transport time to exercise facilities.

### 2.2 Intervention

The study consisted of a 10‐week normal daily lives control period followed by a 10‐week supervised exercise period with walking or running 3/week for 30 min, consisting of 5 min warm‐up and 25 min moderate aerobic intensity of 70–80% of maximum heart rate (HR) on an anti‐gravity treadmill (AlterG Anti‐Gravity Treadmill M320).

### 2.3 Outcome measures

The primary outcome was the 6MWT. The secondary outcomes were (1) postural stability test—overall (PST; Biosway Portable Balance System 950‐460), (2) clinical test of sensory integration and balance (CT‐SIB; Biosway Portable Balance System 950‐460), (3) fatigue severity scale (FSS), (4) short form (36) health survey (SF‐36), (5) Berg balance scale (BBS), and (6) fitness (Berthelsen et al., 2014).

### 2.4 Statistical analyses

Sample size calculations were performed using PS: Power and Sample Size Calculation version 3.1.2, 2014 (Dupont & Plummer, 1990). Statistics were performed in SPSS Statistics 22. Parametric and nonparametric analyses were applied as appropriate for intention‐to‐treat (ITT) and per protocol (PP) analyses. ITT analysis was performed on the patients from Table 1 who had more than one exercise session. The alpha level was *p* ≤ .05 (see Data S1).

**Table 1:** Patient characteristics

Patient/subtype	Sex/age/BMI	Smoking/use of aid	Sensory symptoms	Muscle strength (Nm) (hip flex/ext, knee flex/ext, ankle plantarflex/dorsalflex)	Physical activity (hours last 7 days)^a^	Status
Patient 1/CMT1A	F/62/27	N/none	AB toes^b^^,^ c ^,^ d ^,^ e	31/58, 32/60, 6/0	0/0/0/91	Drop out
Patient 2/CMT1A	M/53/28	N/none	AB toes^b^^,^ c ^,^ d ^,^ e	33/37, 25/69, 48/0	0/0/6/49	Completed
Patient 3/CMTX	M/64/21	N/ankle foot orthoses	NA^b^^,^ c ^,^ d, AB SIAS^e^	46/66, 44/118, 0/0	3/1/7/28	Completed
Patient 4/CMT1A	F/62/28	N/orthopedic shoes	AB toes^b^^,^ c ^,^ d, AB crus^e^	29/65, 26/58, 7/0	0/ND/5.25/ND	Completed
Patient 5/CMT1A	M/31/34	N/insoles	AB toes^b^^,^ c, NA^d^, AB malleolus^e^	93/168, 90/163, 49/29	0/2.3/1.5/56	Completed
Patient 6/CMT1A	F/50/28	N/orthopedic shoes	NA^b^^,^ c, AB toes^d^, AB knee^e^	53/147, 58/121, 13/6	0.5/2/4.5/70	Completed
Patient 7/CMTX	M/46/23	Y/ankle foot orthoses	AB toes^b^, AB knee^c^, AB malleolus^d^, AB SIAS^e^	84/124, 64/196, 8/0	0//0/7/84	Drop out

F, female; M, male; BMI, body mass index (kg/m^2^); Y, yes, N, no; NA, nothing abnormal; AB, abnormal; ND, not determined; SIAS, Spina iliaca anterior superior; Nm, Newton meter.

a Exhaustive exercise/moderate exercise/walking/sitting.

b Proprioception.

c Sensation.

d Pain.

e Vibration.

### 2.5 Ethics

The study was approved by the Capital Region Committee on Health Research Ethics (H‐2‐2014‐095), and written informed consent was obtained.

## 3 RESULTS

Patient characteristics are described in Table 1. Ten patients were recruited, and five patients withdrew (see Data S2). One patient lost balance during anti‐gravity exercise, but completed the exercise. No serious adverse events were reported. There was no significant difference between completers and dropouts regarding age, body mass index (BMI), and muscle strength (*p* ≥ .267). Based on sample size calculations the study was underpowered (see Data S2).

**Figure 1:**
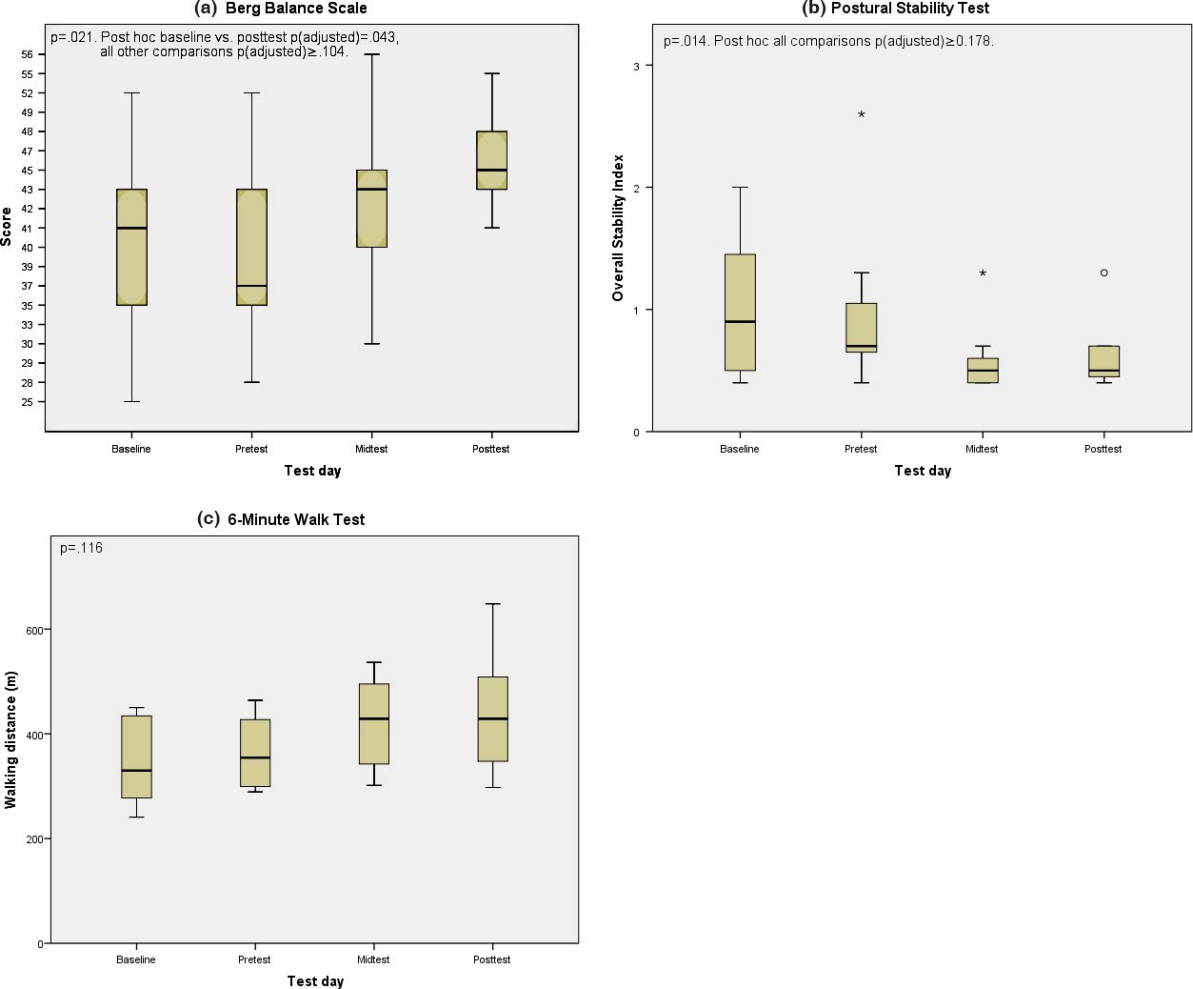
Berg balance scale (BBS), postural stability test (PST), and 6‐min walk test (6MWT) at test occasion (intention‐to‐treat [ITT]). Circle and asterisk are outliers 1.5–3.0 and >3.0 box length from edge, respectively

ITT analysis showed a statistically significant difference between baseline, pretest, midtest, and posttest for BBS and PST, but 6MWT only trended to increase (Figure 1, Table 2). The other outcomes did not change significantly (see data for all outcomes in Table 3).

**Table 2:** Change in 6MWT, BBS, and PST between test occasions (ITT)

	6MWT^a^	BBS^b^	PST^b^
Baseline–pretest	17 ± 17	0 (−6;8)	0 (−.5;.3)
Baseline–midtest	71 ± 38	4 (0;5)	−.2 (−.7;0)
Baseline–posttest	92 ± 53	3 (2;12)	−.2 (−.6;0)
Pretest–midtest	54 ± 25	4 (0;5)	−.2 (−.4;0)
Pretest–posttest	75 ± 39	8 (1;10)	−.1 (−.4;0)
Midtest–posttest	21 ± 16	2 (0;6)	0 (0;.1)

6MWT, 6‐min walk test (m); BBS, Berg balance scale (score); PST, postural stability test (index); ITT, intention‐to‐treat analysis.

a Mean ± standard error of the mean.

b Median (25th;75th percentile).

**Table 3:** Primary and secondary outcomes at test occasions (ITT and PP)

	Baseline (ITT/PP)	Pretest (ITT/PP)	Midtest (ITT/PP)	Posttest (ITT/PP)	*p*‐value (ITT)	*p*‐value (PP)
**Primary outcome**						
6MWT (incl. outlier)^a^	349 ± 90/349 ± 90	366 ± 74/366 ± 74	420 ± 93/420 ± 93	441 ± 123/466 ± 137	.116	.119
6MWT (excl. outlier)^b^	376(284;447)/376(241;447)	361(290;446)/361(289;446)	393(321;490)/393(302;490)	398(320;508)/438(298;509)	.160	.058
**Secondary outcomes**						
SF‐36; physical functioning^b^	45(30;60)/45(10;60)	38(33;60)/38(25;60)	50(20;85)/50(10;85)	55(20;90)/70(45;93)	.375	.157
SF‐36; role limitations due to physical health^b^	50(0;100)/50(0;100)	25(0;50)/25(0;50)	75(25;100)/75(0;100)	75(0;100)/100(0;100)	.322	.151
SF‐36; role limitations due to emotional problems^b^	100(100;100)/100(33;100)	100(100;100)/100(100;100)	100(67;100)/100(33;100)	100(100;100)/100(33;100)	.261	.392
SF‐36; energy/fatigue^b^	55(46;65)/55(35;65)	45(30;70)/45(20;70)	65(40;90)/65(20;90)	65(35;85)/70(45;90)	.664	.431
SF‐36; emotional well‐being^b^	76(68;88)/76(52;88)	84(64;96)/84(44;96)	88(68;96)/88(36;96)	84(68;88)/84(48;88)	.774	.924
SF‐36; social functioning^b^	75(50;100)/75(25;100)	88(63;100)/88(50;100)	100(50;100)/100(50;100)	100(50;100)/100(63;100)	.636	.392
SF‐36; pain^b^	68(45;100)/68(33;100)	58(45;90)/58(45;90)	78(45;90)/78(23;90)	78(45;100)/90(58;100)	.943	.310
SF‐36; general health^b^	51(30;70)/51(5;70)	50(25;60)/50(15;60)	45(35;65)/45(20;65)	50(30;60)/55(25;68)	.909	.259
FSS^b^	4.3(3.5;5.9)/4.3(2.8;5.9)	5.6(4.2;5.9)/5.6(3.3;5.9)	4.4(3.9;5.9)/4.4(3.1;5.9)	4.9(3.0;5.9)/4.2(2.6;4.9)	.303	.238
BBS^b^	41(29;45)/41(25;45)	37(33;48)/37(28;48)	43(37;45)/43(30;45)	45(43;49)/47(43;52)	.021^c^	.121
CT‐SIB; eyes open, firm surface^b^	.52(.35;.93)/.52(.35;93)	.73(.28;.81)/73(.28;.81)	.60(.37;.76)/.60(.37;.76)	.54(.31;.94)/.54(.31;.98)	.766	.782
CT‐SIB; eyes closed, firm surface^b^	1.44(1.17;1.94)/1.44(1.17;1.94)	1.32(1.27;1.80)/1.32(1.27;1.80)	1.69(.82;2.21)/1.69(.82;2.21)	1.48(.78;2.08)/1.35(.78;1.74)	.996	.996
CT‐SIB; eyes open, foam surface^b^	1.29(.90;3.42)/1.29(.90;3.42)	1.40(.88;1.61)/1.40(.88;1.61)	1.02(.84;1.44)/1.02(.84;1.44)	1.04(.71;1.18)/.91(.71;1.08)	.205	.468
CT‐SIB; eyes closed, foam surface^b^	NP	NP	NP	NP	NP	NP
PST; overall^b^	.90(.40;1.80)/.90(.40;1.80)	.70(.40;1.30)/.70(.40;1.30)	.50(.40;.70)/.50(.40;.70)	.50(.40;.70)/.50(.40;.60)	.014^d^	.023^d^
Fitness^b^	NA	118(96;127)/118(96;127)	104(99;128)/104(97;115)	102(98;124)/102(94;120)	.438	.449

ITT, intention‐to‐treat analysis; PP, per protocol analysis; 6MWT, 6‐min walk test (m); SF‐36, short form (36) health survey (score); FSS, fatigue severity scale (score); BBS, Berg balance scale (score); CT‐SIB, clinical test of sensory integration and balance (index); PST, postural stability test (index); NA, not applicable; NP, not possible to complete. Fitness (average heart rate in the fifth minute of moderate‐intensity walking).

a Mean ± 1.96 *SD*.

b Median (25th; 75th percentile).

c *p*‐values are for analyses of variance between more than two repeated measurements. Post hoc baseline–posttest; *p*_adjusted_ = .043, all other comparisons; *p*_adjusted_ ≥ .104).

Post hoc all comparisons; *p*_adjusted_ ≥ .178.

d Post hoc all comparisons; *p*_adjusted_ ≥ .120.

## 4 DISCUSSION

This small pilot study showed a significant positive change in balance and a trend toward improvement in walking capacity with anti‐gravity treadmill training in CMT. This was so, even though a priori power calculation suggested a need for including six patients. Post hoc analysis of BBS showed a significant difference between baseline and posttest only, which indicates that the difference was not solely caused by the intervention. For PST, post hoc analysis was unable to detect where the difference between measurements was. This is probably due to the reduced power of post hoc multiple comparison tests.

ITT and PP analyses showed similar results for all outcomes, except for BBS (ITT *p* = .021, PP *p* = .121). ITT is more conservative, thus, it is more difficult to detect a possible effect, but the result might be explained by dropouts that reduce the power in PP compared to ITT.

One patient increased the walking distance in the 6MWT with approximately 100 m between each test occasion from baseline to posttest, which is a remarkably difference questioning the credibility of the results. The patient's post‐HR after 6MWT was similar for baseline, pre‐, and midtest (post‐HR: 114–117), but increased substantially at posttest (post‐HR: 167). Thus, at least some of the increased walking distance at posttest is explained by a learning effect with greater physical effort at retest. A learning effect for 6MWT has previously been documented for NMDs (Knak, Andersen, Witting, & Vissing, 2017; Prahm, Witting, & Vissing, 2017). Caution should be exercised when applying HR as an indicator of physical effort in training studies, as HR is influenced by fitness, but this is primarily of relevance if HR is reduced after the aerobic intervention, which was not the case in the present patient. Results did not change when the outlier was excluded, although *p*‐value for 6MWT, excluding the outlier, was near .05 in PP analysis.

The temporary pain in two patients might be due to overload, since the patients did not perform regularly aerobic exercise prior to the study, and there was no run‐in period with progression of aerobic intensity.

A study (Wright et al., 1996) in CMT indicated that walking on the ground was well‐tolerated and may improve aerobic capacity, and Maggi et al. (2011) indicated that walking on a normal treadmill combined with stretching, respiratory, and proprioceptive exercises improved walking capacity without overwork weakness in CMT. Berthelsen et al. (2014) showed effect of anti‐gravity strength and aerobic exercises on walking capacity and balance in weak patients with muscular dystrophies. Compared to the present underpowered pilot study, aerobic capacity was not improved, balance assessments showed mixed results, walking capacity indicated a trend toward improvement, and two patients experienced temporary pain. Thus, further studies are needed to assess the true magnitude of the effect of anti‐gravity training in patients with marked muscle weakness.

The study was limited by: (1) lack of sufficient power, introducing a possible type II error, (2) small sample limiting the generalizability, (3) retesting by several investigators, and (4) accessibility of the anti‐gravity treadmill is limited.

In conclusion, our pilot study showed a significant positive change in balance and a trend toward improvement in walking capacity, suggesting that anti‐gravity treadmill training of hereditary neuropathies should be pursued in a larger cohort of patients.

## CONFLICT OF INTEREST

K. L. Knak and L. K. Andersen have received grants from Lundbeckfonden, Rigshospitalets Jubilæumsfond, Vanførefonden, Johannes Fogs Fond, Familien Hede Nielsens Fond, and Grosserer L. F. Foghts Fond. J. Vissing reports no disclosures.

## References

[brb3794-bib-0001] Berthelsen, M. P. , Husu, E. , Christensen, S. B. , Prahm, K. P. , Vissing, J. , & Jensen, B. R. (2014). Anti‐gravity training improves walking capacity and postural balance in patients with muscular dystrophy. Neuromuscular Disorders, 24(6), 492–498. 24684860 10.1016/j.nmd.2014.03.001

[brb3794-bib-0002] Dupont, W. , & Plummer, W. (1990). Power and sample size calculations. A review and computer program. Controlled Clinical Trials, 11(2), 116–128. 2161310 10.1016/0197-2456(90)90005-m

[brb3794-bib-0003] Jani‐Acsadi, A. , Krajewski, K. , & Shy, M. E. (2008). Charcot‐Marie‐Tooth neuropathies: Diagnosis and management. Seminars in Neurology, 28(2), 185–194. 18351520 10.1055/s-2008-1062264

[brb3794-bib-0014] Knak, K. L. , Andersen, L. K. , Witting, N. , & Vissing, J. (2017). The 2‐ and 6‐Minute Walk Tests in Neuromuscular Diseases: Effect of Heart Rate Correction on the Learning Effect. International Journal of Physical Medicine & Rehabilitation, 5(4), https://doi.org/10.4172/2329-9096.1000415. 10.2340/16501977-2222 28352938

[brb3794-bib-0004] Maggi, G. , Monti Bragadin, M. , Padua, L. , Fiorina, E. , Bellone, E. , Grandis, M. , … Schenone, A. (2011). Outcome measures and rehabilitation treatment in patients affected by Charcot‐Marie‐Tooth neuropathy: A pilot study. American Journal of Physical Medicine Rehabilitation/Association of Academic Physiatrists, 90(8), 628–637. 10.1097/PHM.0b013e31821f6e32 21681064

[brb3794-bib-0005] Prahm, K. P. , Witting, N. , & Vissing, J. (2014). Decreased variability of the 6‐minute walk test by heart rate correction in patients with neuromuscular disease. PLoS ONE, 9(12), e114273. 25479403 10.1371/journal.pone.0114273 PMC4257612

[brb3794-bib-0006] Sman, A. D. , Hackett, D. , Fiatarone, S. M. , Fornusek, C. , Menezes, M. P. , & Burns, J. (2015). Systematic review of exercise for Charcot‐Marie‐Tooth disease. Journal of the Peripheral Nervous System, 20(4), 347–362. 26010435 10.1111/jns.12116

[brb3794-bib-0007] Wright, N. C. , Kilmer, D. D. , McCrory, M. A. , Aitkens, S. G. , Holcomb, B. J. , & Bernauer, E. M. (1996). Aerobic walking in slowly progressive neuromuscular disease: Effect of a 12‐week program. Archives of Physical Medicine and Rehabilitation, 77(1), 64–69. 8554477 10.1016/s0003-9993(96)90222-1

