# Supplementary material for: Aerobic anti‐gravity exercise in patients with Charcot–Marie–Tooth disease types 1A and X: A pilot study
Source: Brain Behav. 2017 Nov 2;7(12):e00794. doi: 10.1002/brb3.794 (PMC5745236; doi:10.1002/brb3.794)
Supplement: Supplementary file 1 [file BRB3-7-e00794-s001.docx]

**Supplementary File 1: Statistical analyses**

A priori- and post hoc sample size calculation were performed for the primary outcome, 6MWT, using PS: Power and Sample Size Calculation version 3.1.2, 2014 (1). Since repeated measurement analysis is not an option as a test in PS: Power and Sample Size Calculation, t-test was selected with adjustment by dividing the *p*-value with six comparisons in order to meet the repeated measurement design. A priori sample size calculation was based on the following estimates; (i) Alpha; 0.0083 (p-value 0.05/6 comparisons = 0.0083) (ii) Power; 0.80 (iii) Effect size; 30 m and (iv) Standard deviation (SD); 13 m. The estimates were based on previous studies (2–4). Post hoc sample size calculation was based on the actual standard deviation of the patients recruited for this study. Descriptive and inference statistics were performed in SPSS Statistics 22. Parametric data is presented as mean±1.96SD for descriptive statistics and as mean±1.96SE (standard error of the mean) for inference statistics. Non-parametric data is presented as median (25^th^ percentile, 75^th^ percentile) for descriptive and inference statistics. Data was considered normally distributed if all numeric normality tests were fulfilled; Shapiro-Wilk’s test *p*>0.05 and z-values ±1.96 for Skewness and Kurtosis. Data fulfilling parametric assumptions were assessed by repeated measures one-way analysis of variance (ANOVA) and data not fulfilling parametric assumptions were analysed using Friedman’s two-way analysis of variance (ANOVA) by ranks. Difference in descriptive data between completers and drop outs was evaluated by Mann-Whitney U test. Statistically significant results were analysed with post hoc multiple comparisons tests. Results were considered statistically significant at: (i) *p*≤.05 for repeated measures one-way ANOVA, Friedman’s two-way ANOVA by ranks and for adjusted *p*-values in post hoc analyses and (ii) *p*≤0.008 (*p*=.05/6 comparisons) for post hoc multiple Wilcoxon signed ranks tests. The alpha level for each outcome measure was not adjusted for multiple outcome measures, because there was only one primary outcome measure that was confirmatory. The secondary outcome measures were only explorative and therefore needs to be further investigated in future trials. Thus, a Bonferroni correction for multiple outcome measures would be very conservative. Both Intention-To-Treat (ITT) analysis with “last value carried forward” and Per-Protocol (PP) analysis were performed.

References

1. Dupont W, Plummer W. Power and sample size calculations. A review and computer program. Control Clin Trials. 1990(11(2)):116–28.

2. Henricson E, Abresch R, Han JJ, Nicorici A, Goude Keller E, de Bie E, et al. The 6-Minute Walk Test and Person-Reported Outcomes in Boys with Duchenne Muscular Dystrophy and Typically Developing Controls: Longitudinal Comparisons and Clinically-Meaningful Changes Over One Year. PLoS Curr. 2013;5.

3. McDonald CM, Henricson EK, Han JJ, Abresch T, Nicorici A, Elfring GL, et al. The 6-minute walk test as a new outcome measure in duchenne muscular dystrophy. 2010(41(4)):500–10.

4. van der Ploeg AT, Clemens PR, Corzo D, Escolar DM, Florence J, Groeneveld GJ, et al. A randomized study of alglucosidase alfa in late-onset Pompe’s disease. N Engl J Med. 2010 Apr 15;362(15):1396–406.
