# Supplementary material for: Aerobic anti‐gravity exercise in patients with Charcot–Marie–Tooth disease types 1A and X: A pilot study
Source: Brain Behav. 2017 Nov 2;7(12):e00794. doi: 10.1002/brb3.794 (PMC5745236; doi:10.1002/brb3.794)
Supplement: Supplementary file 2 [file BRB3-7-e00794-s002.docx]

**Supplementary File 2: Reasons for withdrawal and power-calculation**

Ten patients were recruited, and five patients withdrew; two before initiation of the intervention, one after one exercise session and two midway in the intervention. Reasons for withdrawal; (i) Pregnancy (1) (ii) Time constraints (2) (iii) Temporary calf pain (1) and (iv) Temporary hip and back pain (1).

Based on a priori power-calculation six patients were needed according to the following estimates; alpha 0.0083 (p-value 0.05/6 comparisons = 0.0083), power 0.80, effect size 30 m and standard deviation 13 m.

Based on a post hoc power-calculation 112 patients were needed according to the standard deviation of 90 m in the sample and the following estimates; alpha 0.0083 (p-value 0.05/6 comparisons = 0.0083), power 0.80, effect size 30 m.
